# Prohibition of hormones in animal reproduction: what to expect and what to do?

**DOI:** 10.1590/1984-3143-AR2023-0067

**Published:** 2023-09-04

**Authors:** Gabriel Amilcar Bó, Alejo Menchaca

**Affiliations:** 1 Instituto de Reproducción Animal Córdoba, Córdoba, Argentina; 2 Instituto de Ciencias Básicas, Medicina Veterinaria, Universidad Nacional de Villa María, Villa del Rosario, Córdoba, Argentina; 3 Fundación Instituto de Reproducción Animal Uruguay, Montevideo, Uruguay; 4 Plataforma de Investigación en Salud Animal, Instituto Nacional de Investigación Agropecuaria, Montevideo, Uruguay

**Keywords:** estradiol, fixed-time AI, GnRH, eCG

## Abstract

As our understanding of ovarian function in cattle has improved, our ability to control it has also increased. The development of Fixed-Time Artificial Insemination (FTAI) protocols at the end of the 20th century has increased exponentially the number of animals inseminated over the last 20 years. The main reasons for this growth were the possibility of obtaining acceptable pregnancy rates without heat detection and, above all, the induction of cyclicity in suckled cows in postpartum anestrus and prepubertal heifers at the beginning of the breeding season. Most FTAI treatments in South America have been based on the use of progesterone (P4) releasing devices and estradiol to synchronize both follicular wave emergence and ovulation, with pregnancy rates ranging from 40 to 60%. These protocols are implemented on a regular basis, allowing producers access to high-quality genetics, and increasing the overall pregnancy rates during the breeding season. In addition, it provided the professionals involved in these programs with a new source of income and the diversification of their practices into activities other than their usual clinical work. Many of these practices are now apparently at risk from restrictions on the use of estradiol by the European Union (EU) and other countries. However, the development of alternative protocols based on GnRH, with P4 devices and eCG and other new products that are not in the market yet will allow us to adapt to the new times that are coming. Logically, the challenge has already been raised and we must learn to use alternative protocols to try to continue increasing the use of this technology in beef and dairy herds. The objective of the present review is to describe the main aspects of banning estradiol in livestock production, the negative impacts on reproductive efficiency, and to present some alternative FTAI protocols for dairy and beef cattle.

## Introduction

The ban on the use of estradiol in livestock by the European Union (EU) transcends technical reasons linked to bovine reproduction. To understand it, it is convenient to consider the commercial and political interests behind this measure that affects several countries. Estradiol-based drugs (estradiol-17β and its esters) are routinely used in fixed/time artificial insemination (FTAI) programs in cattle in most countries outside Europe, particularly in Latin America. In EU countries its use is not allowed, as well as the importation of food derived from animals that have received estradiol (Council of the European Union, 1981; 1996; 2003), thus exerting pressure to the countries supplying beef and milk to the EU. Consequently, Uruguay suspended in January 2021 the importation, preparation for internal use, commercialization, possession and use of estradiol, and Paraguay, Chile and Argentina have adapted its regulations in recent years. Prohibiting the use of estradiol generates a significant economic loss in these countries generated by a lower use of FTAI, which is reflected in a lower overall pregnancy rate, fewer calves produced, and animals slaughtered.

The EU bases its position mainly on one very specific aspect: food safety. However, this argument by the European authorities lacks support since there is no scientific evidence that shows that the use of estradiol in FTAI protocols generates any risk for consumers. Perhaps for this reason, although the ban on estradiol from the EU was announced several years ago, in the countries of our region there was skepticism that the announcement would materialize, and that this moment would finally arrive. The objective of the present review is to describe the main aspects of banning estradiol in livestock production, the negative impacts on reproductive efficiency, and to present some alternative FTAI protocols for dairy and beef cattle.

## Manipulation of ovarian function for fixed-time AI (FTAI) with estradiol esters

Estradiol valerate was the first estradiol salt used originally at the start of a 9-day norgestomet implant (called Syncro-Mate-B) protocol and was intended to cause uterine-induced luteolysis (Wiltbank et al., 1965). Subsequently, we demonstrated that estradiol valerate additionally suppresses antral follicle growth (Bó et al., 1991). The mechanism of estrogen-induced follicular atresia appears to be systemic and involves suppression of FSH (Bó et al., 1994, 1995). Once the exogenously administered estradiol is metabolized, there is an increase in FSH and a new follicular wave grows (Bó et al., 1994). The administration of 5 mg of estradiol-17β (E-17β; Bó et al., 1994) or 2 mg of estradiol benzoate (EB; Martínez et al., 2005) or estradiol valerate (EV; Colazo et al., 2005) in cattle treated with progesterone (P4) results in the emergence of a follicular wave in 3 to 5 days.

In the synchronization protocols initially developed, 2 mg of EB is administered at the time of insertion of a P4 releasing device that is removed 7, 8 or 9 days later at the time of prostaglandin F2α (PGF2α) administration (Mapletoft et al., 2003; Bó et al., 2013). A 1 mg dose of EB was administered 24 hours later to induce a preovulatory LH surge at 16 to 18 hours (Martínez et al., 2007) and ovulation approximately 30 hours later. This allowed the FTAI with acceptable pregnancy rates (i.e., 40-60%). As an alternative, the administration of 0.5 to 1.0 mg of estradiol cypionate (ECP; Colazo et al., 2003) at the time of P4 device removal is today the most widely used treatment to reduce the number of handlings (Colazo et al., 2003, 2004; Sales et al., 2012; Baruselli et al., 2017; Bó et al., 2019).

During the past two decades, this technology represented the greatest revolution that has occurred in the reproductive management of livestock, with an enormous impact in many countries. Before FTAI, in the 1990s, AI was done on estrus detection and no more than 2 or 3% of the breeding females were inseminated. Currently, more than 30,000,0000 bovine females receive FTAI every breeding season in Brazil, Argentina, Paraguay and Uruguay (Baruselli et al., 2017; Mapletoft et al., 2018; Bo et al., 2018). For the beef and dairy industry, the benefit is such that for every dollar that the producer invests in FTAI, it generates a return of 4 to 5 dollars for the country (Baruselli et al., 2017), which, transferred to the Mercosur scale, represents an annual gross income of more than 2 billion dollars for this region.

## The bad reputation of estradiol

Estradiol plays a fundamental role in FTAI protocols, mainly in cows in anestrus and with low body condition since it has a very precise effect on ovarian control. So why ban it? The greatest difficulty when discussing the use of estradiol in livestock farming arises because two completely different indications for use are considered in the same way: a) estradiol for ovarian control, and b) estradiol as a growth promoter. Estradiol for ovarian control is used at very low doses (0.5 to 2 mg of ECP or EB), intramuscularly and in a single administration. These doses are extremely low in relation to the indicated use as a growth promoter in beef cattle, where it is used in doses 10 to 100 times higher, administered by prolonged-release subcutaneous implants for 60 to 120 days, and generally every animal receives several implants in succession (Smith and Johnson, 2020). This use as a growth promoter has led to questions about the safety of food derived from these animals. Although this has no connection with the use of estradiol in reproduction, the regulatory consequences have been the same. The first contradiction then is that both uses have been considered in the same way and that the same regulatory measures are applied to two absolutely different uses, dosages and formulations.

Estradiol and its derivatives have been used as growth promoters for several decades, long before it was proposed for ovarian control protocols. Already in the 50s of the last century, estradiol derivatives began to be used in the USA in the form of implants in order to improve growth rate in cattle (Dinusson et al., 1950). In the 1880s-1990s, more than 90% of the animals managed in US feedlots received estradiol-based growth-promoting implants and other drugs (Scheffler et al., 2003). The use of these implants improves the growth rate but has also led to questions about the safety of the meat for consumers. In the 1880s, after several years of debate between government authorities from different countries, various commissions and specialized working groups, international organizations, the media and the general public, the EU prohibited the use of those substances used as growth promoters, including estradiol. Shortly after, the importation of food derived from animals that had received these drugs was also banned. The EU measure came into force in 1989. Although in the first instance the European ban did not cover hormones for estrous cycle control, a few years later the regulation was expanded, and estradiol was also prohibited for this purpose.

This measure had global consequences, not only for European producers but also for those countries outside the bloc that supply the EU with meat and milk derivatives. In Uruguay (1988), the use of growth promoters was prohibited in 1988, although the use of hormones to control reproduction was authorized (Decree nº 915/988). Argentina (2004) has similar legislation that has been in force since 2004 (Resolution 447/2004). New Zealand allowed the use of growth promoters (MPI, 2017) although in 2008 it was forced by the EU to prohibit the use of estradiol for the control of estrus. Growth promoters are also available in other countries such as Food Standars Australia and New Zealand, 2021, Canada, South Africa, Japan, while estradiol salts for ovarian control are still available but have undergone certain restrictions. In the USA, growth-promoting implants have been used for more than 60 years and there are more than 30 approved drugs (Smith and Johnson, 2020; FDA, 2023), while estradiol salts in injectable formulations for ovarian control are not available. In short, although the use of estradiol to promote growth remains valid in several countries, its use for ovarian control shows a tendency to be increasingly restricted. As indicated earlier, in South American countries, estradiol has been used for FTAI, embryo transfer and superovulation protocols. However, it is suggested that, as it has happened in several countries (EU, USA, NZ, Uruguay), the farewell to estradiol in reproduction will also reach other countries in the region.

There are no solid arguments that prove that the estradiol used in the doses indicated for FTAI and embryo transfer protocols affects food safety. Furthermore, there is no method available in practice to identify whether a cow received an estradiol dose for FTAI. The reason is that estradiol salts, after being injected, are hydrolyzed to estradiol-17β in a few hours, and this substance is the main estrogen produced naturally by the cow, reaching high levels during estrus or at the end of pregnancy. It is the same molecule that the cow has, and therefore with classical methods such as radioimmunoassay, ELISA or chemiluminescence, it is not possible to identify between exogenous and endogenous estradiol, even when only a few hours have passed since the treatment. In recent years there have been advances combining other more precise methods, such as gas chromatography coupled with isotope ratio mass spectrometry, which seek to identify whether an animal has received synthetic estradiol from the determination of the ratio between the isotopes. Although promising, these methods are complex and expensive, they are not yet fully validated, they are not found in the official residue control programs for estradiol, and they are not effective for the determination of estradiol either, since the dose administered and the concentration in tissues is extremely low, and thus the sensitivity of the method makes the determination very difficult. It should also be considered that a cow that receives estradiol for FTAI or an embryo transfer is normally not sent to the market until after parturition and the calf has been weaned. Even for those that do not become pregnant, it takes several months from treatment to slaughter, because cows need to first being diagnosed as not pregnant and they are then usually fattened. Finally, these animals that go to slaughter at the end of their productive life, are generally old animals or animals that would not meet the conditions for the European market. The problem is that the EU prohibits the use of estradiol at any stage of the animal's life, and no waiting time, maximum residue limit, or acceptable daily intake values are considered for this product, which are the parameters that they are typically used for product registration and risk analysis. This makes it even more difficult to find a fair solution that considers the best interests of both parties.

This article does not intend to discuss in depth the scientific evidence supporting the safety of the use of estradiol for ovarian control. If the reader wishes to learn more about these aspects, specific bibliography can be consulted (Levy, 2010). For reference purposes only, the doses used in FTAI induce circulating estradiol levels similar to those of a cow in estrus 12 to 24 hours after treatment and then return to basal levels a few hours later, with no significant differences to the untreated controls by 48 or 72 hours (Bosolasco et al., 2021). For this reason, EB and ECP do not require a withdrawal period in beef or dairy cattle, as indicated by most of the registries approved by the regulatory agencies of each country outside of Europe. Even though it is used as a growth promoter in much higher doses and for much longer times than for ovarian control, in the USA the FDA does not require a waiting time for implants with estradiol, which indicates that this prestigious regulatory agency does not find any arguments that suggest a risk to consumers FDA, 2023.

While in various instances of the Codex Alimentarius Commission - another world reference body - it is reiterated that there is insufficient evidence to prohibit the use of these substances, the EU argues that the prohibition is based on the fact that the public opposes the use of “hormones” for fattening animals (Codex Alimentarius Comission, 1989, 2001). This then transcends the discussion based on scientific evidence and responds more to a public preference and political reasons. The measure initially taken by the EU in the 1980s arose in the European Parliament, at the suggestion of the European Commission, being advised at that time by the former Scientific Committee on Veterinary Measures Related to Public Health (SCVPH). This scientific committee considered in the analysis studies carried out in conditions very different from the use of estradiol for ovarian control, and they are of very limited value if a conclusion is to be reached for this use. The SCVPH did not consider studies in cattle at low doses as those used for ovarian control, and even so its conclusion on the use as growth promoters is contradictory to the conclusion of all the other independent committees that have acted in different countries. In Great Britain, the Veterinary Products Committee, an independent scientific committee in that country, was asked by the Department of Agriculture, Fisheries and Food to study the evidence of the opinion of the European SCVPH. At the same time, the Safety Working Group of the Committee for Veterinary Medicinal Products responsible vis-à-vis the European Commission for evaluating the safety of veterinary products at the European Medicines Agency (EMA), also studied the opinion of the SCVPH. The British committee made its report in 1999 stating that it did not agree with some of the views of the SCVPH and reported serious doubts in the interpretation that had been made of some of the scientific results. Also in 1999, the EMA committee issued its report EMEA/CVMP/885/99 showing serious differences with the opinion of the SCVPH, and in particular those linked to estradiol. Its conclusions also coincide with those of the FAO/WHO specialist committee reported in 2000 (Joint FAO/WHO Expert Committee on Food Additives, 1999). A few years later, the EU, far from reducing the measure, increased it by prohibiting the use of estradiol for estrus synchronization, and again Great Britain and some other member countries expressed the lack of scientific evidence in the new opinion of the SCVPH (DEFRA, 2006). However, the measure remained in force in 2008 (Directive 2008/97/CE; European Parliament, 2008). In North America, the Center for Veterinary Medicine of the FDA in the US and the Veterinary Drugs Directorate of Canada, the highest regulatory authorities in those countries, maintain dozens of approved products.

In short, the only committee that found some evidence in scientific studies to prohibit the use of estradiol is the European SCVPH, whose opinion coincides with the intention of the EU Commission. However, this interpretation is contrary to the reports of other European committees or working groups in Great Britain or the EMA, as well as in the US, Canada, Codex Alimentarius, FAO/WHO, WTO, among others. Furthermore, the discussion has focused on its use as a growth promoter and similar measures have been taken for ovarian control, when there is no information to suggest that such use could generate any effect on safety.

In summary, the chronicle on estradiol allows us to better understand the reasons that lead countries that supply commodities to take various measures on their production systems. First of all, it is necessary to understand consumer trends and consider them in our agendas. We must be proactive in generating scientific evidence that provides sufficient support to establish a system that is appropriate for consumers and fair for food providers. It is necessary to develop capacities to generate alternatives to our farming practices to adapt them to the needs of the market, even when these respond to commercial or cultural interests or simply to consumer preferences. It is of little use that our animals live according to their natural condition in the field, feeding on grass and in the open air, without suffering, preserving the environment and generating healthy and safe food, if we are not capable of measuring it, making it known and defending it. For this, it is necessary that we understand the need to invest resources in this important issue and do it together, researchers, science and technology institutions, authorities, government agencies, veterinarians, livestock producers and industry.

## Recent studies on estradiol-free protocols

### Gonadotropin-releasing hormone (GnRH)-based protocols in *Bos taurus* beef cattle

Probably the best-known alternative to estradiol-based protocols for FTAI are those based on GnRH. Pursley et al. (1995) have developed an ovulation synchronization protocol for FTAI in lactating dairy cattle that uses GnRH, a protocol called Ovsynch, which with several modifications is today the most widely used protocol in dairy cattle in the world (Consentini et al., 2021). In beef cattle, GnRH-based protocols are used in North America and Europe, and for a couple of years in Uruguay, due to the restrictions imposed by the EU. The first modification of the original OvSynch protocol used in dairy was to simplify it, by placing the second GnRH at the time of the FTAI (protocol called Co-Synch; Geary et al., 2001). In addition, Co-Synch protocols include the insertion of a P4 releasing device in heifers (Martínez et al., 2002) and in postpartum anestrous cows (Lamb et al., 2001).

GnRH-based protocols in beef cattle have evolved over the years and new prolonged proestrus protocols were developed, with the aim of increasing the period of preovulatory exposure to estradiol and improving uterine function and early embryo development (Bridges et al., 2008, 2012, 2014). Also, the greater exposure to preovulatory estradiol was related to lower embryonic losses in the period of time between the maternal recognition of pregnancy and the adhesion of the placental membranes (Madsen et al., 2015). The protocol was called the 5-day Co-Synch+P4 and resulted in grater pregnancy rates than the 7-day Co-Synch+P4 in beef cows (Bridges et al., 2008; Whittier et al., 2013).

Due to the shorter interval between the first GnRH and the induction of luteolysis in the 5-day Co-Synch+P4 protocol, it is recommended to use two doses of PGF2α in cows with an interval of 6 to 24 h (Souto et al., 2009). In an experiment with 2,465 postpartum beef cows, the pregnancy rate was greater in cows that received 2 PGF2α 8 hours apart (55%) than in those that received only one PGF2α (48%), and those that received 2 PGF2α administered at the same time had an intermediate pregnancy rate (51%; Bridges et al., 2012). Therefore, double PGF2α administered 8 to 24 h apart appears to be necessary to maximize fertility with the 5-day protocol in cows. However, if herd management conditions do not allow for further manipulation, a double dose of PGF2α administered at device removal would be an acceptable alternative.

In heifers, the 5-day Co-Synch+P4 protocol has also been tested with modifications (Day, 2015); for example, Colazo and Ambrose (2011) and Cruppe et al. (2014) showed that pregnancy rate did not differ in heifers that did not receive GnRH at the time of P4 device insertion. However, other authors found different results (Kasimanickam et al., 2014). Also in heifers, some found higher pregnancy rates when two doses of PGF2α were used with intervals between 6 and 24 h (Peterson et al., 2011; Lima et al., 2013; Day, 2015), while others did not report differences (Rabaglino et al., 2010; Kasimanickam et al., 2014; Garcia Pintos et al., 2022;). In relation to the optimal time for FTAI, Kasimanickam et al. (2012) reported a greater pregnancy rate with Angus heifers inseminated at 56 h after device removal than those inseminated at 72 h and Day (2015) suggested performing the FTAI at 60 to 66 h after P4 device removal or to inseminate 12 h post estrus using patches or paint and inseminate and administer GnRH to all those not in heat at 72 h. Indeed, estrus expression has been shown to influence pregnancy rates in cows (Richardson et al., 2016), and Colazo et al. (2017) have reported similar findings in heifers inseminated with sexed semen; suggesting the possibility of dividing the insemination based on the expression of estrus (ie, delaying insemination in those animals that do not show estrus at the time of FTAI).

**Figure 1:**
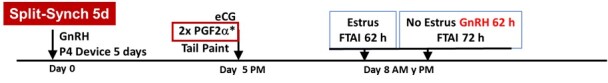
5-day Split-Synch protocol for cows. *In heifers, the second PGF injection could be omitted and the recommended eCG dose is 200 IU [adapted from Garcia Pintos et al. (2022)].

The 5-day Co-Synch protocol has also been extensively evaluated in Uruguay (Garcia Pintos et al., 2022), where it was also decided to slightly alter the original 5-day Co-Synch+P4 protocol by modifying the time for device removal and the FTAI (protocol called 5-day Split-Synch). In this way, the administration of PGF2α is facilitated, by giving the first PGF2α when the device is removed and the eCG is administered on Day 5 PM, then the cows remain in the corrals overnight and receive the second PGF2α before releasing them to the pasture in the morning. Furthermore, tail-paint is also placed at the base of the tail to detect those that are already in heat at the time of the first insemination, which is performed from approximately 62 h after P4 device removal (i.e. Day 8 AM). Those that are not in estrus by that time (i.e. with <50% of the tail-paint rubbed off) receive a GnRH injection and are inseminated in the afternoon (Figure 1).

The same protocol was used in heifers, and it was reported that in this category only one dose of PGF2α is necessary at the end of treatment to induce luteolysis. Most important of all, the results in heifers were similar to those obtained with the J-Synch protocol and in cows they were similar to those obtained with the conventional estradiol-based protocol, with ECP as an ovulation inducer. With this scheme it is possible to inseminate animals all day, thus allowing the implementation of FTAI programs on a large scale, without negatively affecting pregnancy rates.

## GnRH-based protocols in *Bos indicus* beef cattle

The 5-day Co-Synch+P4 protocol has also been investigated in *Bos indicus* cows in Brazil, with a lower pregnancy rate in suckled Nelore cows than those treated with the conventional 8-day estradiol-based protocol (Ferraz et al., 2016). One important difference was that 400 IU eCG was used in the estradiol and P4 based protocol, but not in the 5-day Co-Synch+P4 protocol. To confirm this notion, we have found a greater pregnancy rate in postpartum anestrus cows that received 400 IU eCG upon P4 device removal (5-day Co-Synch+P4: 46.3%, 120/259) than in cows treated with the 5-day Co-Synch+P4 protocol, but without eCG (26.8%, 71/265; P <0.05; Huguenine et al., 2013). In turn, the pregnancy rates of the Co-Synch protocol plus eCG and the conventional estradiol-based protocol with ECP were not different (46.3% for the Co-Synch+P4+eCG protocol and 54.5% for the protocol with EB+P4+ECP+ eCG, respectively).

Also, we recently conducted an experiment to evaluate pregnancy rates to FTAI in *Bos indicus* crossbred cows synchronized with Co-Synch protocols (Bó et al., 2023). Cross-bred *Bos indicus* mature suckled cows (n = 1,161), that were 60 to 90 d postpartum, with a CL or at least one follicle ≥ 8 mm in diameter (detected by ultrasonography) and a body condition score between 2 and 4 (scale 1 to 5) were randomly allocated into one of three groups: J-Synch 7 d, Co-Synch 6 d, and Co-Synch 5 d. On Day 0 (i.e., random day of the estrus cycle), cows in the J-Synch 7 d group received 2 mg of EB (Gonadiol, Zoetis, Ecuador) and an intravaginal device with 0.5 g of P4 (DIB 0.5 g, Zoetis, Ecuador). Cows in the two Co-Synch groups received 100 µg of gonadorelin (GnRH, Gonasyn GDR, Zoetis, Ecuador) on Day 1 (Co-Synch 6 d) or on Day 2 (Co-Synch 5 d), respectively. On Day 7, all cows received 500 µg of cloprostenol sodium (PGF2α; Ciclase DL, Zoetis, Ecuador), 300 IU of equine chorionic gonadotropin (eCG; Novormon 5000, Zoetis, Ecuador), with the difference that cows in the two Co-Synch groups also received a second dose of PGF2α 8 h later. In addition, all the cows were tail painted for estrus determination at the time of FTAI. Cows that had their paint loss ≥50% by 70 h after P4 device removal were inseminated at that time and cows without their tail-paint rubbed off received GnRH at that time and were inseminated 6 to 8 h later. The percentage of cows in estrus at AI was 68.1% (791/1161) and did not differ among groups (J-Synch 7 d: 70.1% 291/415, Co-Synch 6 d: 68.0% 252/370 and Co-Synch 5 d: 66.0% 248/376). However, pregnancy rate was greater in cows in the J-Synch 7 d group (55.0% 228/415) than those in the Co-Synch 6 d (45.0% 167/370) and Co-Synch 5 d (38.5% 145/376) groups. In summary, the estradiol-based synchronization protocol (J-Synch) resulted in greater P/AI than the two GnRH-based protocols evaluated in suckling *Bos indicus* beef cows. Lower pregnancy rates in the *Bos indicus* cows may be attributed to the lower effectiveness of GnRH than EB in synchronizing the emergence of a new follicle wave, and it may be necessary to double the dose of GnRH, since it has been reported that the magnitude of the LH surge produced by the administration of GnRH is lower in *Bos indicus* than in *Bos taurus*, especially in cows with a CL (Batista et al., 2017). Furthermore, it has been shown recently that duplicating the dosage of the GnRH analog buserelin (i.e. 8,4 vs 16,2 µg) significantly increased ovulation rate in Bos indicus cows, but not in heifers with high circulating progesterone concentrations (Silva et al., 2020). Obviously further studies are required to thoroughly investigate this issue.

## Long GnRH+P4-based protocols

One of the main limitations for the application of protocols with GnRH in beef cows and heifers is the low response to the first dose of GnRH (Geary et al., 2000; Martinez et al., 2000). Recently, Bonacker et al. (2020b) developed a new synchronization protocol called 7 & 7 Synch, using previous knowledge generated by Small et al. (2009). This protocol consists of applying PGF2α and a P4 device on Day -7 as a pre-synchronization treatment to develop a persistent follicle; on Day 0, GnRH is administered on Day 0 to ovulate to the persistent follicle and synchronize the emergence of a new follicular wave; on Day 7, all cows received PGF2α and device removal and finally all cows are FTAI, with a dose of GnRH, 60 to 66 hours after device removal. The 7 & 7 Synch protocol demonstrated an improvement in the ovulatory response to the first GnRH administration (Bonacker et al., 2020b) and pregnancy rates to FTAI were increased, both with conventional semen and with sexed semen, when compared against a 7-day Co-Synch treatment + P4 in suckled beef cows (Andersen et al., 2021). In addition, it was an interesting alternative in recipients transferred at a fixed time with fresh and frozen embryos, improving estrus, utilization and pregnancy rates per synchronized recipient, when compared to the 7-day Co-Synch protocol (Bonacker et al., 2020a).

**Figure 2:**
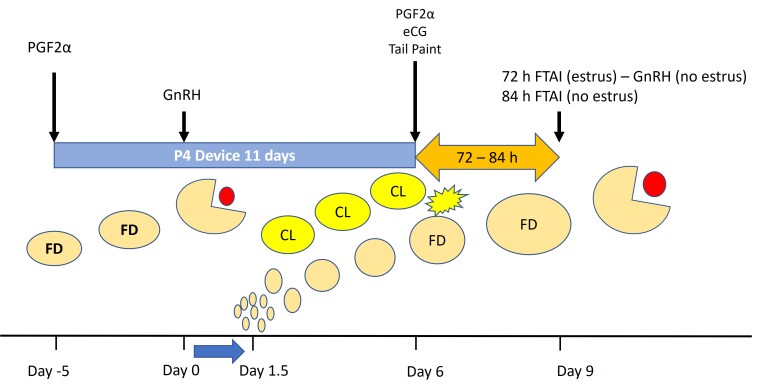
Schematic representation of the Web-Synch protocol. Although a new device with >1 g of P4 must be used in dairy cows, the preliminary evidence indicates that in beef cows a device with 0.6 or 0.7 g of P4 may be used. In addition, the treatment in lactating dairy cows includes a second injection of PGF2α on Day 7. FD: Dominant follicle

Based on this knowledge and given the restrictions on the use of estradiol in EU certified farms in Argentina, an alternative treatment based on GnRH was designed, which we called “Web-Synch” (Without Estradiol Benzoate). Briefly, this treatment is a slight modification of the 7 & 7 Synch (de la Mata et al., 2022). On Day -5 a pre-synchronization treatment is initiated with the administration of PGF2α and a P4 device, to generate a persistent follicle. On Day 0, GnRH is injected to induce ovulation of the persistent follicle and promote the emergence of a new follicular wave (36 hours later). Subsequently, on Day 6, the device is removed along with a dose of PGF2α and eCG, to induce follicular growth and promote a prolonged proestrus (as in the 5-day Co-Synch treatments) and tail-paint is used for estrus detection. Finally, FTAI is performed 72-84 h after P4 device removal, with the application of GnRH only to animals that are not in estrus by that time (Figure 2).

In three experiments carried out to evaluate the Web-Synch protocol in suckled beef cows, the time of ovulation and pregnancy rates to AI did not differ with those of cows treated with the J-Synch protocol (de la Mata et al., 2022). Both the estrus rate and the pregnancy rate did not differ between treatments, being 66.0 and 58.7% for Web-Synch, and 58.0 and 54.1% for J-Synch (P>0, 1), respectively. Furthermore, no differences in pregnancy rates were detected between cows treated with a 1 g P4 device or a 0.7 g of P4 device (Sincrover, Laboratorios Over SRL, Argentina).

In this sense, more recent experiments were recently carried out in beef and dairy cows but comparing the treatment with the conventional estradiol-based protocol (i.e. 2 mg EB at P4 device insertion and 1 mg ECP at P4 device removal). In Dairy cows 79% of the cows ovulated to the first GnRH in the Web-Synch protocol and the pregnancy rate was greater (P<0.01) in those of the Web-Synch group (52.9%, 136/262) than in those of the Conventional group (37.8% 102/270) respectively (Macagno et al., 2022).

In beef cows the results were somewhat contradictory, with similar pregnancy rates between cows treated with the Web-Synch protocol (44.3%, 39/88) than in those treated with the conventional (45.1%, 37/82; P=0,3) in cows with a moderate to high incidence of cyclicity (i.e. 51% of the cows with a CL on Day 0; de la Mata et al., 2023a). However, in another group of cows in which only 9.8% of them had the CL on Day 0, pregnancy rate was greater in those receiving the conventional treatment (66.3%, 102/154) than those receiving the Web-Synch protocol (49.4%, 79/160; P=0,01; de la Mata et al., 2023b). Although it was not evaluated in these studies, we speculate that differences may be due to a lower ovulation rate to the first GnRH in anestrus beef cows or to differences in the uterine environment due to lower estradiol in the proestrus period in the cows not treated with ECP at P4 device removal.

In conclusion, the Web-Synch protocol could be considered as a new alternative for synchronization of ovulation and FTAI. Certainly, the results have been promising in dairy cows and in beef cows with moderate to high incidence of cyclicity. More studies must be done to further evaluate the reason for lower pregnancy rates in herds with greater incidence of postpartum anestrus. It is also noteworthy, that the benefits to the application of this protocol must be important in order to justify the extra handling in beef cattle. One final comment, although GnRH is today the best-known alternative to estradiol, we must keep our minds open to other options that may efficiently synchronize follicular wave emergence in cattle for FTAI and fixed-time embryo transfer.

## Equine Chorionic Gonadotropin (eCG)

A final paragraph is included to briefly mention the use of eCG. In its natural form, this hormone is a high molecular weight glycoprotein produced by the endometrial cups of the mare's uterus between 35 and 100 days of gestation and it is extracted from the blood of pregnant mares, which have raised some animal welfare concerns in some European countries. In the mare, eCG has LH activity, but in the cow, eCG can have either FSH or LH activity, depending on the receptor populations in the ovarian follicles at the time. Although eCG was originally used to induce superovulation, today its use in cattle is more oriented towards stimulating the growth of the dominant follicle that results in greater pregnancy rates in FTAI and Fixed-time embryo transfer (FTET; Bó et al., 2002, 2016, 2019; Baruselli et al., 2004, 2012, 2017; Nuñez-Olivera et al., 2014; Randi et al., 2021).

Although until recently 100% of the eCG used in bovines was produced through the bleeding of pregnant mares, today we have the possibility of producing this hormone in the laboratory. These hormones are generically called “recombinant”. Today we have at least one recombinant eCG in the Argentine market and the experiments recently carried out showed that the addition of recombinant eCG increases the pregnancy rate in suckled cows (Villarraza et al., 2021). Other recombinant eCG are about to appear on the market with similar results (Abreu et al., 2022) and it is expected that in the future recombinant and/or synthetic hormones will replace those obtained from animals, due to political pressure from the environmental groups.

## Final comments

Undoubtedly, the advance in the knowledge of the reproductive physiology of the cow will allow us to face the next challenges in the implementation of reproductive technologies in beef and dairy cattle. Our obligation will always be to maximize productivity in order to produce food at low cost for a growing population, but we need to do that by creating efficient protocols and management strategies minimizing the negative impact to the environment. Furthermore, we must educate the general public about it and show that we can feed the world with safe methodologies, with animal welfare and taking care of the environment.
